# Climate change, cash transfers and health

**DOI:** 10.2471/BLT.14.150037

**Published:** 2015-05-20

**Authors:** Frank Pega, Caroline Shaw, Kumanan Rasanathan, Jennifer Yablonski, Ichiro Kawachi, Simon Hales

**Affiliations:** a Department of Public Health, University of Otago, Wellington, PO Box 7343, Wellington, New Zealand.; b United Nations Children's Fund, New York, United States of America (USA).; c Department of Social and Behavioral Sciences, Harvard TH Chan School of Public Health, Boston, USA.

## Abstract

The forecast consequences of climate change on human health are profound, especially in low- and middle-income countries and among the most disadvantaged populations. Innovative policy tools are needed to address the adverse health effects of climate change. Cash transfers are established policy tools for protecting population health before, during and after climate-related disasters. For example, the Ethiopian Productive Safety Net Programme provides cash transfers to reduce food insecurity resulting from droughts. We propose extending cash transfer interventions to more proactive measures to improve health in the context of climate change. We identify promising cash transfer schemes that could be used to prevent the adverse health consequences of climatic hazards. Cash transfers for using emission-free, active modes of transport – e.g. cash for cycling to work – could prevent future adverse health consequences by contributing to climate change mitigation and, at the same time, improving current population health. Another example is cash transfers provided to communities that decide to move to areas in which their lives and health are not threatened by climatic disasters. More research on such interventions is needed to ensure that they are effective, ethical, equitable and cost–effective.

## Résumé

Les conséquences attendues du changement climatique sur la santé humaine sont importantes, en particulier dans les pays à revenu faible et intermédiaire et pour les populations les plus défavorisées. Des moyens d'intervention innovants sont nécessaires pour lutter contre les effets néfastes du changement climatique sur la santé. Les transferts d'argent sont des moyens d'intervention éprouvés pour protéger la santé de la population avant, pendant et après les catastrophes climatiques. Le Programme de création de dispositifs de sécurité productifs de l'Éthiopie, par exemple, prévoit des transferts d'argent pour réduire l'insécurité alimentaire découlant des périodes de sécheresse. Nous proposons d'inclure les opérations de transfert d'argent dans des actions plus préventives en vue d'améliorer la santé dans le contexte du changement climatique. Nous avons identifié différents systèmes de transfert d'argent prometteurs qui pourraient être utilisés pour éviter les conséquences néfastes des risques liés au climat sur la santé. Les transferts d'argent visant l'utilisation de moyens de transport actifs et sans émissions – pour se rendre au travail à vélo par ex. – pourraient prévenir les futures conséquences néfastes sur la santé en contribuant à l'atténuation du changement climatique et en améliorant ainsi l'état actuel de la santé de la population. Un autre exemple concerne les transferts d'argent accordés aux communautés qui décident de s'établir dans des régions où leur vie et leur santé ne sont pas menacées par des catastrophes climatiques. Davantage de recherches sur ces opérations sont nécessaires pour prouver leur efficacité, leur caractère éthique et équitable ainsi que leur rentabilité

## Resumen

Las consecuencias previstas del cambio climático en la salud humana son severas, especialmente en los países de ingresos bajos y medios y entre los grupos más desfavorecidos. Se necesitan instrumentos normativos innovadoras para afrontar los efectos adversos sobre la salud que el cambio climático produce. Las transferencias de efectivo son instrumentos normativos establecidos para proteger la salud de la población antes, durante y después de los desastres relacionados con el clima. Por ejemplo, el Programa “Red de Seguridad Productiva” de Etiopía proporciona transferencias de efectivo para reducir la inseguridad alimentaria derivada de las sequías. Nosotros proponemos extender las intervenciones de transferencias de efectivo a medidas más proactivas para mejorar la salud en el contexto del cambio climático. Identificamos planes prometedores de transferencia de efectivo que podrían utilizarse para prevenir las consecuencias adversas sobre la salud provocadas por los riesgos climáticos. Las transferencias de efectivo para usar modos de transporte activos y libres de emisiones (por ejemplo, dinero para ir al trabajo en bicicleta) podrían prevenir futuras consecuencias adversas sobre la salud, contribuyendo a la mitigación del cambio climático y, al mismo tiempo, mejorando la salud actual de la población. Otro ejemplo son las transferencias de efectivo realizadas a comunidades que deciden trasladarse a zonas dónde sus vidas y su salud no estén amenazadas por los desastres climáticos. Es necesario llevar a cabo más investigaciones en estas intervenciones para garantizar que sean efectivas, éticas, equitativas y costoefectivas.

## ملخص

إن للتبعات المنظورة لتغير المناخ والتي تمس صحة الإنسان آثارٌ شديدة الخطورة، خاصةً في البلدان منخفضة ومتوسطة الدخل وفي القطاعات السكانية المحرومة. لذا فقد اقتضت الحاجة وجود أدوات مبتكرة لسياسة التصدي للآثار الضارة المترتبة على تغير المناخ فيما يتعلق بالصحة. وتمثل التحويلات النقدية أدوات متبعة للسياسة المعنية بحماية صحة السكان قبل وأثناء وبعد وقوع الكوارث المتعلقة بالمناخ. فعلى سبيل المثال، يقدم برنامج شبكات الأمان الإنتاجية الإثيوبي تحويلات نقدية للتقليل من انعدام الأمن الغذائي الناتج عن موجات الجفاف. ونقترح توسيع نطاق تدخلات التحويل النقدي لتشمل إجراءات تتسم بدرجة أعلى من المبادرة لتحسين الصحة في إطار تغير المناخ. وقد حددنا خططًا واعدة للتحويل النقدي يمكن استخدامها لمنع الآثار الضارة بالصحة والمترتبة على الأخطار المناخية. ويمكن للتحويلات النقدية المخصصة لاستخدام وسائل مواصلات فعالة لا تصدر انبعاثات ضارة – مثل النقد المخصص لاستخدام الدرجات للانتقال إلى العمل – أن تمنع الآثار الصحية الضارة في المستقبل، وذلك من خلال المساهمة في الحد من تغيرات المناخ وفي تحسين صحة السكان الحالية في نفس الوقت. ومثال آخر على ذلك التحويلات النقدية المقدمة للمجتمعات التي قرر أفرادها الانتقال إلى مناطق لا تمثل فيها الكوارث المناخية تهديدًا لأرواحهم أو صحتهم. وتقتضي الحاجة إجراء المزيد من الأبحاث على هذا النوع من التدخلات لضمان أنها فعالة ومراعية للمعايير الأخلاقية ومنصفة ومجدية التكلفة.

## 摘要

气候变化对人类健康具有可预测的深远影响，在中低收入的国家和大部分弱势群体中尤为如此。 我们需要使用创新的政策工具来应对气候变化对健康造成的不利影响。 现金资助是已经确立的政策工具，可在与气候有关的灾害发生之前、期间或之后保护全民健康。 例如，埃塞俄比亚的“生产安全网计划”提供现金资助，可降低因干旱造成的粮食不安全性。 我们建议将现金资助干预措施拓展为更加积极的措施，在气候变化的环境下改善健康状况。 我们确定了有价值的现金资助方案，可以用来预防气候灾害对健康造成的不利影响。 为采用零排放、积极的交通模式提供现金资助（例如，骑车上班的现金资助）有助于减缓气候变化，可预防今后对健康造成不利影响，同时还能改善当前的全民健康状况。 另外一个示例是，向决定搬迁到新地区的社区团体提供现金资助，在那里他们的生活和健康不会受到气候灾害的威胁。 我们需要针对该种干预措施开展更多研究，以确保其有效性、伦理性、公平性和成本效益。

## Резюме

Прогнозируемые последствия изменения климата для здоровья человечества являются значительными, особенно в странах с низким и средним уровнем доходов и среди наиболее уязвимых слоев населения. Для преодоления негативного влияния климатических изменений на здоровье людей необходимы новые политические инструменты. Одним из таких инструментов, уже показавших свою эффективность в плане защиты здоровья населения до стихийных бедствий, вызванных климатическими изменениями, во время и после них, являются денежные переводы. Например, денежные переводы используются в рамках программы Productive Safety Net в Эфиопии для снижения риска нехватки продовольствия в результате засухи. Мы предлагаем не ограничиваться мерами по переводу денежных средств и дополнить их более активными упреждающими мероприятиями, направленными на улучшение здоровья людей в контексте изменения климата. Мы определяем перспективные схемы перевода денежных средств, которые могут использоваться для предотвращения неблагоприятных для здоровья последствий стихийных бедствий. Денежные переводы для использования экологически чистых, активных видов транспорта (например, денежное поощрение за использование велосипеда, чтобы добраться до работы) могут предотвратить негативные последствия в будущем, способствуя смягчению изменений климата и одновременно улучшая здоровье населения. Другим примером могут служить денежные переводы общинам, которые решили переехать в регионы, где стихийные бедствия не будут угрожать их здоровью и жизни. Необходимо провести дополнительные исследования, чтобы обеспечить эффективность, этичность, справедливость и экономичность подобных мероприятий.

## Introduction

Innovative policy tools are needed to address the adverse consequences of climate change.^1^ So-called cash transfers are established policy tools for protecting population health before, during and after climate-related disasters.^2^^,^^3^ Here we propose the extension of cash transfer schemes to more proactive measures for improving health in the context of broader climate change and social protection policies and systems.

## Climate change and health

In 2014, a report of the Intergovernmental Panel on Climate Change (IPCC) stated that:

“Without additional efforts to reduce greenhouse gas emissions beyond those in place today, emissions growth is expected to persist driven by growth in global population and economic activities. Baseline scenarios, those without additional mitigation, result in global mean surface temperature increases in 2100 from 3.7 to 4.8 °C compared to pre‐industrial levels.”^1^

The probable adverse health consequences of a 3.7–4.8 °C increase in global mean surface temperature are profound. The direct consequences include higher morbidity and mortality from extreme weather events such as heat waves, droughts and floods.^1^ Between 2030 and 2050, increased heat exposure alone is forecast to cause 38 000 additional deaths per year among older people.^4^ Compared with the direct effects, the indirect health effects are likely to be even more substantial and more difficult to predict and quantify.^5^ The effects of climate change on ecosystems may lead to increased exposure to vector-borne and waterborne diseases and changes in water distribution and availability.^6^ Other effects on human life may include loss of livelihoods, food insecurity, population migration, displacement and violent conflict.^6^ Between 2030 and 2050, climate change-related increases in diarrhoea, malaria and childhood malnutrition are predicted to result in 203 000 additional deaths per year.^4^

Compared with high-income countries, low- and middle-income countries have higher baseline disease burdens, lower resilience and weaker health and welfare systems and will, in consequence, find it more difficult to cope with the additional burdens imposed by climate change.^1^ Within countries, disadvantaged populations – e.g. poor people and marginalized children – face disproportionate health risks from climate hazards.^1^

The complex adverse health consequences of climate change require intersectoral action. Although several sectors have already combined their efforts against climate change and its consequences, the speed and efficiency of such efforts need to be advanced. Policy tools that improve outcomes from several sectors – e.g. those that improve economic, health and environmental outcomes – are likely to play a crucial role in strengthening global support for such intersectoral action. The lessons learnt from intersectoral action on health equity,^7^ although still limited,^8^ may provide important pointers.

Broadly speaking, interventions for addressing the adverse consequences of climate change can be differentiated into those that mitigate impact– i.e. by reducing the net emissions of so-called greenhouse gases – and those that facilitate adaptation – i.e. by helping populations and at-risk groups to survive in a more hostile environment.^9^ In reality, mitigation and adaptation policies often overlap. For example, if we adapt to heat waves by redesigning urban environments, we may also help to reduce emissions. Both types of policy may also have health co-benefits. For example, by reducing car use to cut emissions, we may also improve population health by improving air quality and levels of physical activity.^10^^,^^11^ However, either type of policy may also bring some adverse health effects, particularly for marginalized communities.^12^^,^^13^ For example, if policies are not carefully designed, the use of market mechanisms or taxation to reduce emissions can reduce low-income groups’ access to essential services.

## Cash transfers

### Improving health

Social protection systems are established components of social policy in most high-income countries.^14^ The well-established welfare systems in Scandinavia, for example, take a rights-based approach to comprehensive social protection. Low- and middle-income countries are now rapidly developing social protection systems.^15^ Many countries that do not yet have comprehensive social protection systems have established – or are currently developing – social protection floors – i.e. nationally defined sets of basic social security guarantees for preventing or alleviating poverty, vulnerability and social exclusion.^16^

National social protection systems in many countries are based on cash transfers.^2^^,^^3^ For example, the flagship of the rapid expansion of Brazil’s social protection system was the introduction of the *Bolsa Família* cash transfer programme in 2003.^17^ Cash transfers are non-contributory cash payments provided by governmental, international and nongovernmental organizations generally to support recipients’ consumption and so address poverty, vulnerability and/or other constraints on human development.^2^^,^^3^ At the most basic level, the economic rationale of regular, ongoing cash transfers is that they ensure a minimum income for individuals, families and communities.^18^ In the long term, this guaranteed minimum income is intended to reduce poverty and foster human development – including better health.^18^ A recipient may also be given one cash transfer or just a few such transfers to buffer the effects of adverse shocks over the short-term – e.g. the income shocks arising from loss of employment or an economic crisis or the income, health or other shocks arising from a humanitarian disaster.^18^ Cash transfers are increasingly seen as a viable alternative to in-kind transfers, where recipients receive either a good or asset other than cash, or a service.

Most cash transfers address key social determinants of health, including health-related environmental factors such as access to safe water and food security.^2^^,^^3^ In some cases, cash transfers are also linked, as part of broader social protection systems, to health insurance schemes and other programmes or policies designed to increase access to health services. Such transfers are often targeted according to poverty or vulnerability criteria but some are universal.^2^^,^^3^ They can be conditional – with a requirement for some specified behaviour – or they can be unconditional – given without obligation.^2^^,^^3^ *Bolsa Família* is a targeted conditional cash transfer programme, where cash transfers are only paid to low-income families if the children in the families are being vaccinated and attending school.^19^ A nearly-universal and unconditional cash transfer is the South African Child Support Grant.^20^

Cash transfers have considerable potential to improve health.^21^^,^^22^ In high-income countries, cash incentives have been used sporadically, with mixed success, to reward healthy behaviours or decrease vulnerability in areas such as drug use, cancer screening and nutrition.^23^ If these incentives are modified appropriately (e.g. targeted to poor people) they can provide promising designs for genuine cash transfer interventions. In low- and middle-income countries however, the use of cash transfers to improve health, particularly among mothers and children, is more common and their effectiveness is better established. In 2012, a systematic review of 13 conditional cash transfer programmes found strong evidence that such transfers improved the use of health services – including preventive services – and health outcomes –including nutritional status, anthropometric measures and prevalence of diseases – among children and adults.^24^

### Addressing climate change

We propose that, by building on broader policies on climate change and social protection systems, cash transfers could be used as a policy tool to address the adverse health consequences of climate change. Such transfers could contribute to the Sustainable Development Goals,^25^ by bringing together economic, health, social and environmental objectives. In the past, action towards individual Millennium Development Goals has often been dominated by single sectors working independently. If the Sustainable Development Goals follow a more integrated, intersectoral approach, cash transfers could help address some of the challenges inherent to reconciling poverty reduction, health improvement, social protection and action on climate change.

Awareness and knowledge about the uses of cash transfers for improving global environmental health are currently limited, even though cash transfers may be more cost–effective than other interventions such as in-kind transfers. Cash transfers were not included in a recent systematic review of public health interventions for reducing the adverse health consequences of climate change^26^ and were mentioned only briefly by the IPCC.^1^ Cash transfers have the potential to protect and improve health in the context of climatic disasters, since they can improve health outcomes before disasters, can increase individual and household capacities to deal with adverse shocks and can buffer shocks in general. However, we focus specifically on designs for cash transfer schemes in the context of climate change and health.

We propose two main types of cash transfers for addressing the health impacts of climate change (Table 1). The first type is designed to prevent adverse health consequences and includes cash transfers that mitigate climate change, allow human populations to adapt to climate change and/or harness the health co-benefits of mitigation or adaptation policies. The second type of cash transfer is designed to manage the adverse health consequences of climate change and includes cash transfers that allow human populations to prepare for future adverse health consequences and/or deal with any existing adverse health consequences.

**Table 1:** Types, subtypes, objectives and examples of cash transfers for addressing the adverse health consequences of climate change

Type of cash transfer	Subtype of cash transfer	Objective	Example of cash transfer
To prevent adverse health consequences	To mitigate climate change	To reduce net greenhouse gas emissions	For installing and using systems that generate renewable energy^a^
		To resettle citizens of areas under threat from climate change	For resettling to areas not threatened by climate-related events
	To adapt to climate change	To adapt to water shortage during a drought	For growing drought-resistant rice varieties and using water-preserving irrigation strategies
		To adapt to climate change in urban contexts	For establishing and operating planted roof tops that collect and purify rainwater for domestic purposes
	To harness health co-benefits of climate change mitigation or adaptation policies	To promote specific policies that reduce emissions and improve health	For using emission-free, active modes of transport^b^
			For retrofitting insulation^c^
To manage adverse health consequences	To prepare for future adverse health consequences of climate change	To prepare for food or water insecurity caused by climate-related events	For seasonal food insecurity due to climate variability^d^
	To deal with existing adverse health consequences of climate change	To improve access to health services and health outcomes by improving income before, during and/or after a climate-related disaster	For people affected by a climate-related disaster^e^

^a^ As implemented – as a non-targeted economic incentive – in the United Kingdom’s Renewable Heat Incentive programme.^27^

^b^ As piloted –as a non-targeted economic incentive – in a French programme of incentives for cycling to work.^28^

^c^ As implemented in Warm Up New Zealand: Healthy Homes scheme.^29^

^d^ As implemented in the Ethiopian Productive Safety Net Programme.^30^

^e^ As implemented in the Nicaraguan *Atención a Crisis* programme.^31^

### Prevention

#### Climate change mitigation 

Cash transfers that mitigate climate change prevent negative health consequences for the general global population, even if they have no immediate health co-benefits. For example, Australia, China, India, Japan and the United Kingdom of Great Britain and Northern Ireland have introduced cash incentives to encourage households to install and operate photovoltaic roof panels, wind turbines or other systems for the generation of renewable energy. The government of the United Kingdom recently introduced long-term cash incentives for installing and using heating systems powered by renewable energy.^27^ Although cash incentives for climate change mitigation are currently generally provided as universal subsidies, they could be disbursed specifically to poor or vulnerable populations as a form of social protection. Similarly, cash transfers could be used as a reward for energy conservation, resulting from the installation of energy-efficient electrical technology.

#### Adaptation to climate change

Cash transfer interventions that ensure better adaptation to climate change should reduce the adverse health consequences of such change. For example, in the agricultural domain, cash could be disbursed to poor farmers so that they can change to more adaptive practices of crop production. In the central lowlands of China, for example, cash transfers could be used to encourage the production of drought-resistant varieties of rice using water-preserving irrigation strategies.^32^ Cash transfers targeted at poor farmers could support income security and enable the farmers to adapt to climate change and increase food security when water supplies become limited.

#### Harnessing health co-benefits

Some cash transfers may more directly and immediately improve health by harnessing the health co-benefits of mitigation or adaptation policies. For example, cash transfers could be designed to motivate uptake of household energy interventions with established health co-benefits through the supply of more energy-efficient houses.^33^ For example, the retrofitting of household insulation can reduce emissions cost–effectively – by reducing energy consumed for heating – and also reduce the incidence of asthma.^34^^,^^35^ Adherence to sustainability codes such as the Passive House Standard^36^ may also reduce emissions and provide immediate health benefits by improving the indoor air quality. However, further research and refinement of such standards may be required.^37^ If such energy efficiency interventions are proven to be robust, their uptake could be encouraged with cash transfers that have the combined aims of strengthening social protection, improving health and tackling climate change.

It is possible to modify roads and paths in ways that facilitate the use of bicycles or other active modes of transport and then use cash incentives to promote the use of such modes of transport by local residents. Cash incentives for cycling to work, for example, already exist in Belgium, Denmark and France. The French government piloted an incentive scheme in which recipients were paid the equivalent of 0.34 United States dollars for each kilometre that they cycled to work.^28^ Cash incentives to off-set the costs of bicycle purchase are another design option. Again, cash transfers could be targeted at poor and vulnerable populations.

### Management

#### Preparation for the future

Several cash transfers that prepare the recipients better for the adverse health consequences of climatic hazards already exist.^38^ Although such transfers can be targeted at the groups that are most vulnerable to climate change, there may be problems in determining the optimal size, timing, focus and structure of these transfers.^38^

In a few countries, cash transfers have already been used to increase resilience against the adverse health consequences of climate change. In Kenya, cash transfers are given to families to ensure that orphans and other vulnerable children attend school and health check-ups.^38^ During severe droughts, such transfers were found to reduce the proportion of children aged 6–13 years who did paid and unpaid work by 4% and 10%, respectively^39^ – i.e. they protected families from having to send their children to work to compensate for income lost because of the droughts. The Kenyan Hunger Safety Net Programme provides regular and predictable cash transfers in response to climate hazards – such as droughts – and their effects – such as crop failures.^38^

Further cash transfer schemes could be developed for communities that decide to move to areas in which their lives and health are less threatened by climate change. Cash transfers could be used to support communities that want to resettle from areas that become uninhabitable because of climate change. Cash transfers might also be used to retrain individuals whose traditional livelihoods are threatened by climate change. For example, fishermen may need retraining when marine ecosystems no longer support fishing. Such schemes require safeguards that prevent communities and individuals from being forced to make changes against their will.

#### Dealing with existing problems

In climate-related disasters, cash transfers are established policy tools. In 2012, the government of Niger, the United Nations Children’s Fund and other partners provided unconditional cash transfers to families with vulnerable children to address food insecurity and broader needs resulting from severe drought. In the Philippines, unconditional cash transfers were used in the aftermath of typhoon Haiyan in 2013.^40^

One randomized controlled trial^31^ and one controlled before-and-after study^41^ have examined the effect of cash transfers for humanitarian assistance in climate-related disasters on health service use and health outcomes. Both studies investigated the use of cash transfers during droughts. According to this small body of evidence cash transfers can not only improve the use of preventive health services but also reduce deaths and disease and improve nutrition among children.

## Effectiveness, ethics and equity

Several issues regarding the general use of cash transfers for improving health remain unresolved.^42^ In terms of effectiveness, there are concerns about the impact of cash transfers on behavioural motivation and the relative effectiveness of each of the different policy designs related to such transfers. Some behavioural theorists worry that conditional cash transfers could undermine any intrinsic motivation for healthy behaviours, ultimately reduce the motivation for healthy behaviours – i.e. after the financial reward has ceased – and/or create an expectation of rewards for other behaviours.^43^ So far, however, empirical studies have failed to provide any justification for such concerns.^44^

Among several debated features of the design of cash transfer interventions, the relative effectiveness of conditional cash transfers compared with unconditional ones^45^ and of cash transfers paid to mothers compared with those paid to fathers,^46^ remains unclear. In the absence of detailed cost–effectiveness analyses of the health impact of the different types of cash transfer designs, it is not clear that the additional administrative costs incurred by attaching conditions to cash transfers are warranted.

Diverse and complex ethical concerns also exist, especially for conditional cash transfers. Many people view the attachment of conditions to cash transfers – that contribute towards fulfilment of the right to social protection^47^ – as paternalistic and stigmatizing, because it assumes that recipients require state-regulated incentives to adopt health-beneficial behaviours.^42^ In contrast, unconditional cash transfers do not carry the same level of ethical concern because they assume that recipients are rational actors who do not require external conditions for them to engage in such healthy behaviours.^46^ Conditionality seems inappropriate in humanitarian settings, both ethically and practically.

Cash transfers that disproportionately improve the most disadvantaged populations’ health are expected to improve health equity in the population.^22^^,^^48^ If, however, the barriers to receiving them are greater for the most disadvantaged, then cash transfers may also decrease health equity. For example, if parents do not conform to the conditions attached to a cash transfer, then their children may be denied benefits through no fault of their own. However, a recent systematic review found no evidence to indicate that cash transfer schemes increased health inequity.^49^

## Research and evaluation

The administrators of cash transfer schemes have gained considerable experience with cash transfers and some have described the lessons they have learnt.^50^ There is some evidence that cash transfers are effective in improving health, but more and better evidence is still needed.^24^ Evidence on cost–effectiveness, ethics and equity impact of cash transfers for improving health remains weak. Apart from the two studies^31^^,^^41^ on cash transfers used in droughts – there appears to be no high-quality evidence of the usefulness of cash transfers for addressing the adverse health consequences of climate change. The focus of most early research on cash transfers was on poverty reduction rather than health improvement. Robust evaluations of the impact of cash transfer schemes are often difficult to establish.^2^

We propose the following research agenda to tackle the evidence gap regarding the use of cash transfers to address the adverse health effects of climate change. First, existing data should be mined to establish the effectiveness, cost–effectiveness, ethics and equity impact of previous and ongoing cash transfer schemes, where feasible. Second, the evaluation systems for the ongoing schemes should be strengthened.^2^ Third, if existing data do not provide sufficient evidence, it may be necessary to design and trial new cash transfer interventions and to evaluate them to determine the optimal design and maximize the potential synergies between economic, health, social and environmental objectives. More research on the feasibility, implementation and sustainability of cash transfer schemes also seems warranted. Any research should be grounded in behavioural theory and explore the potential ethical and equity issues associated with such schemes.

## Conclusion

There are several promising cash-transfer interventions for addressing the adverse health consequences of climate change. Additional research on these interventions is required, to ensure that they are effective, ethical, equitable and good value for money.
